# Statistical analysis of longitudinal data on tumour growth in mice experiments

**DOI:** 10.1038/s41598-020-65767-7

**Published:** 2020-06-04

**Authors:** Ioannis Zavrakidis, Katarzyna Jóźwiak, Michael Hauptmann

**Affiliations:** 1 Department of Epidemiology and Biostatistics, Netherlands Cancer Institute, Amsterdam, Netherlands; 2 Brandenburg Medical School Theodor Fontane, Institute of Biostatistics and Registry Research, Neuruppin, Germany

**Keywords:** Cancer models, Statistical methods

## Abstract

We consider mice experiments where tumour cells are injected so that a tumour starts to grow. When the tumour reaches a certain volume, mice are randomized into treatment groups. Tumour volume is measured repeatedly until the mouse dies or is sacrificed. Tumour growth rates are compared between groups. We propose and evaluate linear regression for analysis accounting for the correlation among repeated measurements per mouse. More specifically, we examined five models with three different variance-covariance structures in order to recommend the least complex method for small to moderate sample sizes encountered in animal experiments. We performed a simulation study based on data from three previous experiments to investigate the properties of estimates of the difference between treatment groups. Models were estimated via marginal modelling using generalized least squares and restricted maximum likelihood estimation. A model with an autoregressive (AR-1) covariance structure was efficient and unbiased retaining nominal coverage and type I error when the AR-1 variance-covariance matrix correctly specified the association between repeated measurements. When the variance-covariance was misspecified, that model was still unbiased but the type I error and the coverage rates were affected depending on the degree of misspecification. A linear regression model with an autoregressive (AR-1) covariance structure is an adequate model to analyse experiments that compare tumour growth rates between treatment groups.

## Introduction

Animal experiments are an invaluable tool for biomedical research, because they allow evaluation of hypotheses by randomization of nearly identical subjects, they can usually be conducted much faster than corresponding human studies (if those are at all ethically feasible), and biological mechanisms in animals are often, however not always, similar to those in humans. Nevertheless, animal studies require careful design and state-of-the-art statistical analysis to ensure robust conclusions with proper control of type I and II error, efficient use of resources, and justifiable use of animals.

Guidelines are available for design, statistical analysis and reporting of animal experiments (ARRIVE^1^), and the UK-based National Center for Replacement, Refinement & Reduction of Animals in Research (http://www.nc3rs.org.uk) provides various resources. These guidelines describe the general principle of conducting studies, ethical conditions in working with animals but also statistical considerations. In a recent series of articles in prominent journals, a plea was made to raise attention to the design and analysis of animal experiments in order to improve the outcome of biomedical research (http://www.nature.com/news/web-tool-aims-to-reduce-flaws-in-animal-studies-1.19459, http://www.nature.com/news/poorly-designed-animal-experiments-in-the-spotlight-1.18559, http://www.nature.com/news/uk-funders-demand-strong-statistics-for-animal-studies-1.17318^2–4^.

Despite the availability of guidelines, the design, statistical analysis and reporting of animal experiments need improvement. A recent survey found, that more than 95% of 48 studies did not report on statistical power and 55% of 180 studies used inappropriate statistical methods^5,6^. Underpowered studies may fail to detect an effect that truly exists or observe an effect larger than the true effect^7,8^. On the other hand, overpowered studies might detect a small effect which is not relevant. In both cases, researchers may report erroneous conclusions and waste animal lives, time and money. Most importantly, follow-up studies, such as clinical trials, might fail because they are based on incorrect assumptions^8–13^.

A commonly investigated outcome is tumour growth after treatment induction. For example, patient-derived tumour xenografts (PDX) are an important preclinical tool for cancer biomarker discovery and drug development. During such longitudinal experiments, animals are injected with human tumour cells and treated after the tumour reaches a certain volume. Tumour size is measured several times per week. Many investigators compare average tumour size between treatment groups at arbitrary time points and therefore ignore the majority of the data. These separate tests have lower statistical power in comparison to a method that uses all of the available data and individual changes within mice are not taken into account while they are accounted for in methods that use all repeated measurements within mice. The importance of the issue was recently highlighted by a report comparing separate analyses at individual time points with analyses of all repeated measurements together in preclinical animal experiments^14^. The authors concluded that the latter indeed yields higher statistical power for detecting a treatment effect and maximally exploits data obtained from animals used in research experiments, which is an ethical obligation. In addition, as Heitjan *et al*.^15^ have shown, performing tests at arbitrary time points leads to inflated type I errors because multiple testing is performed. Linear regression using all of the available data, instead, should be used to estimate tumour growth over time per treatment group and compare the rate of growth between groups. Statistically, this is assessed by an interaction term of time and treatment group. In these models, there are several ways to incorporate the dependence between repeated tumour size measurements within a mouse. If this dependence is not taken into account, point estimates and standard errors of regression coefficients may be incorrect leading to incorrect conclusions with respect to the effect of treatment.

The use of regression methods for the analysis of longitudinal data has been a topic of active research for many years^16,17^, and several articles have investigated the application of these models to small studies in general^18–20^, and to mice experiments of tumour growth in particular^15,21–27^. However, many of these articles described complicated models, and only one article evaluated properties of estimates of the interaction term for small to moderate sample sizes^22^, which is relevant for tumour growth experiments. Our aim is to evaluate several methods to handle the dependence of repeated tumour size measurements within mice in a linear regression setting for the comparison of tumour growth, in order to recommend an easy to use method that is appropriate for small to moderate sample sizes. We perform a simulation study based on data from three previous experiments to investigate the properties of estimates of the treatment group by time interaction term which addresses the difference in tumour growth between two treatment groups.

## Methods

### Data from previous tumour growth experiments

We used data from three previous tumour growth experiments conducted in collaboration with researchers from The Netherlands Cancer Institute. In these experiments, length and width of tumours were measured with a digital calliper 1–3 times per week and tumour volume was calculated as 0.5 × length (in mm) × width (in mm)^2^. These experiments have been published and are briefly described below.

#### DNA damage tolerance (DDT) deficiency in lobular breast carcinoma treated with cisplatin

Buoninfante *et al*.^28^ evaluated the sensitivity of two mammary tumour cell lines, one DDT-proficient, DDT^p^ *(Wap–Cre;Cdh1*^*F/F*^*;SB;Pcna*^*K164*^) and the other DDT-deficient, DDT^D^ (*Wap–Cre;Cdh1*^*F/F*^*;SB;Pcna*^*K146R*^), to cisplatin. Tumour cells were transplanted orthotopically into the fat pad of the mammary gland of NMRI mice. When tumours reached a volume of 100 mm^3^, both groups of mice were treated with cisplatin (6 mg/kg). Mice were killed either when tumour volume exceeded 1,500 mm^3^ or when the tumour had metastasized and the animal was severely distressed.

#### Treatment of cervical cancer with an AXL antibody

Boshuizen *et al*.^29^ studied the anti-tumour activity of the antibody-drug conjugate AXL-107-MMAE in patient-derived xenografts, including melanoma, lung, pancreas and cervical cancer. Nude mice were inoculated subcutaneously at the right flank with one tumour fragment (2–3 mm diameter). Before treatment, mice were divided into groups of 6–8 mice each, with equal tumour size distribution (average and variance). Randomization occurred in a blinded fashion. Mice were treated intraperitoneally or intravenously with solutions containing the AXL-107-MMAE antibody in two different doses as well as several control antibodies, adjusted to actual body weight, according to the schedule specified at each experiment. The experiment ended for individual mice either when the tumour size exceeded 1500 mm^3^, the tumour showed ulceration, the mouse was seriously ill, tumour growth blocked the movement of the mouse, or end of study after 60 days. For this report, we focused on data from xenograft tumour model CV1664 for cervical cancer and treatment by the antibody-drug conjugate AXL-107-MMAE 2 mg/kg and the unconjugated isotype control antibody IgG1-b12 4 mg/kg.

#### Inhibition of SHP2 in KRAS-mutant non-small cell lung cancer

RAS mutations are frequent in human cancer, especially in pancreatic, colorectal and non-small-cell lung cancers (NSCLCs). Mainardi *et al*.^30^ focused on SHP2 (also known as PTPN11) to inhibit the RAS oncoproteins. Wild-type and PTPN11-knockout cells of the AZD6244 (selumetinib)-resistant lung cancer cell line H2122 were injected subcutaneously into the right flanks of 8-week-old immunocompromised CD1 nude female mice. Mice were randomized when the tumour reached a volume of approximately 200–250 mm^3^. AZD6244 was administered daily by oral gavage for a 34-day period. The control group was treated at the same schedule with the vehicle of AZD6244. For this report, we used the data on the H2122 wild-type cells only.

### Statistical analysis

To evaluate whether the rate of tumour growth differs between two treatment groups, we used the linear regression model:

1 $${\log }_{10}{y}_{ij}=\alpha +{\beta }_{1}{t}_{i(j-1)}+{\beta }_{2}{x}_{i}{t}_{i(j-1)}+{\epsilon }_{ij},$$

where ${y}_{ij}$ was the tumour volume of the *i*-th mouse (*i* = 1, …, n) at the *j-*th measurement (*j* = 1, …, *m*), ${x}_{i}$ indicated the treatment of the *i*-th mouse (${x}_{i}=0$ for treatment A, ${x}_{i}=1$ for treatment B) and ${t}_{i(j-1)}$ was the time since randomization of the *i*-th mouse at the *j-*th measurement (${t}_{i0}\,$represented time of the first measurement and ${t}_{i(m-1)}$ represented time of the *m-*th measurement). Since at the time of randomization average tumour volume was expected to be the same between treatment groups, a term representing the average difference in volume at baseline between treatment groups, i.e., the main treatment effect, was omitted from the model. ${{\epsilon }}_{ij}$ was a normally distributed residual for the *j*-th measurement of the *i*-th mouse with expectation zero and variance ${\sigma }^{2}$, i.e., ${{\epsilon }}_{ij} \sim N(0,{\sigma }^{2})$, and the *m* residuals for mouse *i* were stacked into a vector ${{\epsilon }}_{i}=({{\epsilon }}_{i1},\,{{\epsilon }}_{i2},\,\ldots ,\,{{\epsilon }}_{im})\text{'}$ which had a multivariate normal distribution with a vector of *m* zeroes as mean and variance-covariance matrix ${\Sigma }_{i}$, i.e., ${{\epsilon }}_{i} \sim N(0,{\Sigma }_{i})$. Log-transformed tumour volume was used as the outcome to ensure normally distributed residuals and homogeneity of variance over time. We assumed that the number of measurements was the same for each mouse and the association between time and tumour volume on the logarithmic scale was approximately linear. Parameters α, $\,{\beta }_{1}$ and ${\beta }_{2}$ did not vary by mouse. The intercept α denoted the overall average log-volume at the time of randomization, ${\beta }_{1}$ was the linear change in log-volume across time for treatment A, while ${\beta }_{2}$ was the difference between the linear change in log-volume across time between treatment A and B. Thus, a statistical test of the null hypothesis ${\beta }_{2}=0$ addressed the main question whether the tumour growth rates differed between the two treatment groups.

The variance-covariance matrix of the full vector with all residuals ${{\epsilon }}_{ij}\,$in the data had a block structure with a separate block for each mouse, i.e.

$$\Sigma ={\sigma }^{2}[\begin{array}{ccc}\begin{array}{c}\begin{array}{cc}{\Sigma }_{1} & 0\end{array}\\ \begin{array}{cc} & {\Sigma }_{2}\end{array}\end{array} & \cdots  & \begin{array}{c}0\\ 0\end{array}\\  & \ddots  & \vdots \\  &  & {\Sigma }_{n}\end{array}]$$

Since all matrices in this report were symmetric, we only provided the cell entries above the diagonal. We assumed that all ${\Sigma }_{i}$ were identical. In order to accommodate possible dependence between longitudinal measurements, we evaluated the following three different variance-covariance structures of matrix ${\Sigma }_{i}$.

The first model assumed an ***independent (IND)*** variance-covariance structure of matrix ${\Sigma }_{i}$ which had the form:

$${\sum }_{i}={\sigma }^{2}[\begin{array}{cccc}1 & 0 & \ldots  & 0\\  & 1 &  & 0\\  &  & \ddots  & \vdots \\  &  &  & 1\end{array}]$$

All observations in the data were assumed to be independent, even measurements on the same mouse.

The second model used a ***compound symmetry (CS)***, also called exchangeable, variance-covariance structure of matrix ${\Sigma }_{i}$ of the form:

$${\sum }_{i}={\sigma }^{2}[\begin{array}{cccc}1 & \eta  & \ldots  & \eta \\  & 1 &  & \eta \\  &  & \ddots  & \vdots \\  &  &  & 1\end{array}]$$

where $\eta$ was the correlation among measurements within each mouse. This correlation was assumed to be the same for any pair of measurements from the same mouse.

The variance-covariance structure of matrix ${\Sigma }_{i}$ of the third model had an ***autoregressive (AR-1)*** form:

$${\Sigma }_{i}={\sigma }^{2}[\begin{array}{ccccc}1 & {\rho }^{{t}_{i1}-{t}_{i0}} & {\rho }^{{t}_{i2}-{t}_{i0}} & \cdots  & {\rho }^{{t}_{i(m-1)}-{t}_{i0}}\\  & 1 & {\rho }^{{t}_{i2}-{t}_{i1}} & \ldots  & {\rho }^{{t}_{i(m-1)}-{t}_{i1}}\\  &  & 1 & \ldots  & {\rho }^{{t}_{i(m-1)}-{t}_{i2}}\\  &  &  & \ddots  & \vdots \\  &  &  &  & 1\end{array}]$$

where $\rho$ was the correlation between two measurements on consecutive days from the same mouse. The correlation between two measurements decreased as the time difference between them increased.

In the fourth model, the rates of tumour growth between treatment groups were also evaluated using the linear model (1) with the independent variance-covariance structure and an additional dummy variable ${I}_{i}$ indicating observations from mouse *i* (${I}_{i}=1$ for mouse *i* and 0 otherwise; i=1, …, n-1). This model, called a fixed-effects model^31^, had the form:

$${\log }_{10}{y}_{ij}=\gamma +{\beta }_{1}{t}_{i(j-1)}+{\beta }_{2}{x}_{i}{t}_{i(j-1)}+{\beta }_{{3}_{i}}{I}_{i}+{\epsilon }_{ij}$$

One of the mice was chosen to be the reference and $\gamma$ was the log-volume of the tumour of that mouse at randomization. Then, ${\beta }_{{3}_{i}}$ was the difference in log-volume at the time of randomization between mouse *i* and the reference mouse.

As the fifth model, we investigated the linear model (1) with AR-1 variance-covariance structure, which additionally included a random error term for the intercept. This mixed-effects model had the form:

$${\log }_{10}{y}_{ij}=(\alpha +{u}_{0i})+{\beta }_{1}{t}_{i(j-1)}+{\beta }_{2}{x}_{i}{t}_{i(j-1)}+{\epsilon }_{ij},$$

where the term ${u}_{0i}$ represented unexplained variability with respect to the log-volume at the time of randomization between mice. It was assumed normally distributed with zero mean and variance ${\sigma }_{u0}^{2}$, and independent from the error term at the repeated measures level.

Parameters in the four linear regression models were estimated via marginal modelling using generalized least squares (GLS)^32,33^ and restricted maximum likelihood (REML) estimation^34,35^. Estimation of the mixed-effects model was also based on REML.

### Simulation study

We used the third model with an autoregressive (AR-1) variance-covariance structure and empirical data from the three experiments to generate hypothetical data with known effects under realistic circumstances. We generated similar numbers of mice and measurements as in the original experiments. Treatment groups were equally sized and all mice had the same number of measurements, leading to a completely balanced design. For parameters $\alpha$, ${\beta }_{1}$, and ${\sigma }^{2}$ we used values estimated from the original data using GLS and REML with an autoregressive (AR-1) covariance matrix (Table 1). For parameter ${\beta }_{2}$ we used the estimated value and one other value that either reflected a smaller or larger effect than the observed one. For parameter $\rho$ we used the estimated value as well as 0 and 0.5 to evaluate scenarios with uncorrelated and moderately correlated repeated measurements. Therefore, for each experiment, 6 scenarios were simulated (two values of ${\beta }_{2}$ and three values of $\rho$, Table 2). For each scenario, 3000 datasets were generated under a model with an autoregressive covariance matrix. Each dataset was analysed with the five regression models listed above. For each model, the 3000 results were summarized by calculating the average and the first and third quartiles of estimated ${\beta }_{2}$, the proportion of studies where the 95% confidence interval (CI) around the estimate of ${\beta }_{2}$ included the true value (coverage), and the proportion where the 95% CI around the estimate of ${\beta }_{2}$ did not include zero (statistical power). For ${\beta }_{2}=0$, the latter proportion was the type I error. Type I error and coverage were considered nominal if close to 0.05 and 95%, respectively.

**Table 1:** Results of statistical analysis of three tumour growth experiments.

	DDT deficiency (Buoninfante *et al*.)^28^	AXL antibody (Boshuizen *et al*.)^29^	SHP2 inhibition (Mainardi *et al*.)^30^
Treatment groups	WT & K164R	lgG1-b12 4 mg/kg & AXL-107-MMAE 2 mg/kg	AZD6244 & Vehicle
Number of mice/group	15 & 15	6 & 6	7 & 10
Average number of measurements/mouse	18 & 21	16 & 17	8 & 6.2
α (95% CI)	1.982 (1.933, 2.031)	2.115 (1.839, 2.392)	2.433 (2.332, 2.534)
${\beta }_{1}$ (95% CI)	0.025 (0.023, 0.028)	0.016 (0.009, 0.022)	0.017 (0.013, 0.020)
${\beta }_{2}$ (95% CI)	−0.0096 (−0.011, −0.007)	−0.022 (−0.030, −0.014)	−0.008 (−0.012, −0.003)
σ (95% CI)	0.174 (0.158, 0.191)	0.487 (0.342, 0.691)	0.213 (0.168, 0.270)
ρ (95% CI)	0.852 (0.819, 0.880)	0.990 (0.980, 0.995)	0.969 (0.946, 0.982)

Abbreviation: CI, confidence interval.

Note: A linear model $lo{g}_{10}{y}_{ij}=\alpha +{\beta }_{1}{t}_{i(j-1)}+{\beta }_{2}{x}_{i}{t}_{i(j-1)}+{\epsilon }_{ij}$ with an autoregressive (AR-1) covariance matrix was used. α denotes the overall average log-volume at the time of randomization, ${\beta }_{1}$ is the linear change in log-volume across time for the reference group (WT, IgG1-b12 4 mg/kg, Vehicle), while ${\beta }_{2}$ is the difference between the linear change in log-volume across time between the reference group and a comparison group (K164R, AXL-107-MMAE 2 mg/kg, AZD6244), ${{\epsilon }}_{ij} \sim N(0,{\sigma }^{2})$ and ρ is the autocorrelation between adjacent measurements.

**Table 2:** Results of simulation study for the DDT deficiency experiment with 15 mice per group and 18 measurements per mouse^28^.

Scenario	ρ	True ${\beta }_{2}$	Model	Mean estimated ${\beta }_{2}$ (IQR)	Coverage	Power	Type I error**
1	0	−0.002	Ind	−0.0020 (−0.0025, −0.0015)	0.9460	0.7307	0.0520
			AR-1	−0.0020 (−0.0026, −0.0015)	0.9490	0.7243	0.0480
			CS	−0.0020 (−0.0025, −0.0015)	0.9360	0.7300	0.0570
			IND-I	−0.0020 (−0.0030, −0.0009)	0.9490	0.2767	0.0520
			Mixed AR-1*	−0.0020 (−0.0026, −0.0014)	0.9567	0.6648	0.0389
2	0	−0.0096	Ind	−0.0096 (−0.0102, −0.0091)	0.9430	1.0000	
			AR-1	−0.0096 (−0.0102, −0.0091)	0.9460	1.0000	
			CS	−0.0096 (−0.0102, −0.0091)	0.9370	1.0000	
			IND-I	−0.0095 (−0.0106, −0.0086)	0.9510	1.0000	
			Mixed AR-1*	−0.0096 (−0.0101, −0.0091)	0.9570	1.0000	
3	0.5	−0.002	Ind	−0.0020 (−0.0027, −0.0014)	0.8760	0.6830	0.1160
			AR-1	−0.0020 (−0.0027, −0.0014)	0.9450	0.5320	0.0457
			CS	−0.0020 (−0.0027, −0.0013)	0.9090	0.5910	0.0840
			IND-I	−0.0020 (−0.0033, −0.0008)	0.8630	0.3350	0.1340
			Mixed AR-1*	−0.0020 (−0.0027, −0.0013)	0.9523	0.4848	0.0429
4	0.5	−0.0096	Ind	−0.0096 (−0.0103, −0.0089)	0.8743	1.0000	
			AR-1	−0.0096 (−0.0103, −0.0089)	0.9510	1.0000	
			CS	−0.0096 (−0.0103, −0.0089)	0.9180	1.0000	
			IND-I	−0.0096 (−0.0108, −0.0083)	0.8760	1.0000	
			Mixed AR-1*	−0.0096 (−0.0103, −0.0089)	0.9550	1.0000	
5	0.85	−0.002	Ind	−0.0020 (−0.0031, −0.0009)	0.6437	0.6337	**0.3560**
			AR-1	−0.0020 (−0.0031, −0.0009)	0.9483	0.2447	**0.0523**
			CS	−0.0020 (−0.0034, −0.0006)	0.6867	0.5040	**0.3070**
			IND-I	−0.0020 (−0.0038, −0.0002)	0.6340	0.4887	**0.3590**
			Mixed AR-1*	−0.0020 (−0.0030, −0.0010)	0.9554	0.2199	**0.0458**
6^**§**^	**0.85**	**−0.0096**	**Ind**	**−0.0096 (−0.0108, −0.0084)**	**0.6240**	**1.0000**	
			**AR-1**	**−0.0096 (−0.0107, −0.0085)**	**0.9413**	**1.0000**	
			**CS**	**−0.0096 (−0.0111, −0.0081)**	**0.6757**	**0.9990**	
			**IND-I**	**−0.0096 (−0.0115, −0.0077)**	**0.6227**	**0.9947**	
			Mixed AR-1*	**−0.0096 (−0.0107, −0.0085)**	**0.9488**	**1.0000**	

Covariance matrix structures include independence (Ind), autoregressive (AR-1) & compound symmetry (CS). IND-I corresponds to the model with independence covariance structure and a mouse indicator (fixed-effects model). Mixed AR-1 corresponds to the mixed-effects model with random intercept.

*The percentage of datasets for which the model did not converge was 1.3, 2.1, 5.5, 9.6, 8.7, 14.7 for Scenario 1, 2, 3, 4, 5, 6, respectively. For the scenarios for type I error evaluation, the associated percentages were 1.4, 5.2 and 6.8 for ρ of 0, 0.5 and 0.85, respectively.

**Type I error is derived from corresponding scenarios with ${\beta }_{2}$ = 0.

^§^Scenarios in bold face reflect parameter values actually observed in the experiment.

Analyses and simulations were performed using R version 3.4.4^36^ including the nlme package^37^ and were verified using STATA version 15^38^.

### Sensitivity analysis

Since the data was generated using the AR-1 variance-covariance matrix, our simulation study results might have favoured the AR-1 model. Therefore, as a sensitivity analysis, we generated data with another variance-covariance matrix. Specifically, we assumed that the correlation between two measurements decayed with increasing time between the measurements, but in contrast to AR-1 where correlation declined quadratically, we used a structure where it declined linearly:

$${\Sigma }_{i}={\sigma }^{2}[\begin{array}{ccccc}1 & \rho -{\rm{\theta }}\,\ast \,|{t}_{i1}-{t}_{i0}| & \rho -{\rm{\theta }}\,\ast \,|{t}_{i2}-{t}_{i0}| & \cdots  & \rho -{\rm{\theta }}\,\ast \,|{t}_{i(m-1)}-{t}_{i0}|\\  & 1 & \rho -{\rm{\theta }}\,\ast \,|{t}_{i2}-{t}_{i1}| & \ldots  & \rho -{\rm{\theta }}\,\ast \,|{t}_{i(m-1)}-{t}_{i1}|\\  &  & 1 & \ldots  & \rho -{\rm{\theta }}\,\ast \,|{t}_{i(m-1)}-{t}_{i2}|\\  &  &  & \ddots  & \vdots \\  &  &  &  & 1\end{array}]$$

Parameter θ defined the slope of the decline with higher values leading to steeper slopes. We used three different values for θ, namely 0.02, 0.05 and 0.08. For ρ, we used the estimated value as well as 0.5 and for all other parameters, we used the same values as in our main simulation study. Therefore, for each experiment, we simulated 12 scenarios since we used three values of θ, two values of ρ and two values of ${\beta }_{2}$.

## Results

### Observed data from previous growth experiments

For the DDT deficiency experiment, data on 585 measurements in 30 mice yielded $\hat{\alpha }=1.98$ ($95 \% \,CI\,1.933,\,2.031$), ${\hat{\beta }}_{1}=0.025$ ($95 \% \,CI\,0.023,\,0.028$), ${\hat{\beta }}_{2}=-0.0096$ ($95 \% \,CI\,-0.011,\,-0.007$), $\hat{\sigma }=0.175$ ($95 \% \,CI\,0.158,\,0.191$) and $\hat{\rho }=0.852$ ($95 \% \,CI\,0.819,\,0.880)$, indicating that tumour size on a logarithmic scale increased under cisplatin treatment by 0.025 mm^3^ per day among DDT-proficient mice and by ${\hat{\beta }}_{1}+{\hat{\beta }}_{2}=$ 0.016 mm^3^ per day among DDT-deficient mice. The difference between these two rates was statistically significant (p < 0.001).

The AXL antibody experiment included 6 mice per group with an average of 17 measurements per mouse. Estimated parameters are $\hat{\alpha }=$ 2.115 ($95 \% \,CI\,1.839,\,2.392$), ${\hat{\beta }}_{1}=0.016$ ($95 \% \,CI\,0.009,\,0.022$), ${\hat{\beta }}_{2}=-0.022$ ($95 \% \,CI\,-0.030,\,-0.014$), $\hat{\sigma }=0.487$ ($95 \% \,CI\,0.342,\,0.691$) and $\hat{\rho }=0.99$ ($95 \% \,CI\,0.980,\,0.995$), indicating that tumour volume on a logarithmic scale increased by 0.016 mm^3^ per day among mice in the lgG1-b12 4 mg/kg group and decreased by $| {\hat{\beta }}_{1}+{\hat{\beta }}_{2}|=0.006$ mm^3^ per day among mice in the AXL-107-MMAE 2 mg/kg group. There was a significant difference between tumour growth in the two treatment groups (p < 0.001).

In the SHP2 inhibition experiment, 17 mice with a total of 118 measurements were used. The parameters of the models were estimated as $\hat{\alpha }=$ 2.433 ($95 \% \,CI\,2.332,\,2.534$), ${\hat{\beta }}_{1}=0.017$ ($95 \% \,CI\,0.013,\,0.020$), ${\hat{\beta }}_{2}=-0.008$ ($95 \% \,CI\,-0.012,\,-0.003$), $\hat{\sigma }=0.213$ ($95 \% \,CI\,0.168,\,0.270$) and $\hat{\rho }=0.96$ ($95 \% \,CI\,0.946,\,0.982$), indicating that tumour volume on a logarithmic scale increased by 0.017 mm^3^ per day among mice in the vehicle group and by ${\hat{\beta }}_{1}+{\hat{\beta }}_{2}=0.009$ mm^3^ per day among mice in the AZD6244 group. The two growth rates were significantly different (p < 0.001).

Note that in all three experiments the autocorrelation $\rho$ was rather high, suggesting that two consecutive measurements from the same mouse were highly correlated.

### Simulated data based on previous growth experiments

The average across estimates of ${\hat{\beta }}_{2}$ from the generated datasets per scenario were almost identical to the true value of ${\beta }_{2}$ for all evaluated scenarios and all 5 models. Therefore, we obtained unbiased estimates for the difference in tumour growth between two different treatments with all 5 models.

For the model with an independent variance-covariance structure, i.e., no correlation between repeated volume measurements ($\rho =0$), coverage was close to 95% and type I error close to 5% for all evaluated ${\beta }_{2}$ values, all three experiments and three of the investigated models. The model with CS showed coverage slightly below 95% and a type I error above 5%, while the AR-1 mixed-effects model with random intercept showed coverage slightly above 95% and type I error below 5%. For non-zero values of $\rho$, the AR-1 model was the only one which retained nominal coverage and type I error in all scenarios. The AR-1 mixed-effects model with random intercept also resulted in nominal Type I error, while for the other 3 models, type I error was seriously inflated. Note that the observed data from the three experiments showed a high correlation between repeated tumour volume measurements, i.e., high values of $\rho$.

For AR-1, the only method controlling the type I error at the nominal level in all scenarios, power was highest for scenarios with a small $\rho$ and a large ${\beta }_{2}$. For scenarios reflecting the actually observed parameter values in previous experiments, estimated power was high except for the SHP2 inhibition experiment where it was 25%.

All results of our simulation study are presented in Tables 2–4. The numbers are based on the 3000 generated datasets for all models except the AR-1 mixed-effects model with random intercept, since this model was not estimable for all datasets. The percentage of datasets for which the model did not converge varied between 1 and 50 depending on the scenario.

**Table 3:** Results of simulation study for the AXL inhibition experiment with 6 mice per group and 15 measurements per mouse^29^.

Scenario	ρ	True ${\beta }_{2}$	Model	Mean estimated ${\beta }_{2}$ (IQR)	Coverage	Power	Type I error**
1	0	−0.01	Ind	−0.0100(−0.0132, −0.0069)	0.9537	0.5720	0.0600
			AR-1	−0.0100 (−0.0132, −0.0069)	0.9567	0.5570	0.0507
			CS	−0.0100 (−0.0133, −0.0068)	0.9247	0.5837	0.0857
			IND-I	−0.0099 (−0.0156, −0.0043)	0.9517	0.2120	0.0593
			Mixed AR-1*	−0.0100 (−0.0131, −0.0068)	0.9628	0.4965	0.0379
2	0	−0.022	Ind	−0.0218 (−0.0249, −0.0187)	0.9467	0.9967	
			AR-1	−0.0219 (−0.0249, −0.0186)	0.9510	0.9963	
			CS	−0.0219 (−0.0250, −0.0186)	0.9163	0.9963	
			IND-I	−0.0218 (−0.0275, −0.0160)	0.9507	0.7207	
			Mixed AR-1*	−0.0219 (−0.0250, −0.0188)	0.9579	0.9908	
3	0.5	−0.01	Ind	−0.0100 (−0.0138, −0.0061)	0.8777	0.5580	0.1157
			AR-1	−0.0100 (−0.0139, −0.0061)	0.9417	0.4073	0.0497
			CS	−0.0100 (−0.0143, −0.0059)	0.8900	0.4783	0.0973
			IND-I	−0.0102 (−0.0175, −0.0030)	0.8627	0.2983	0.1383
			Mixed AR-1*	−0.0100 (−0.0138, −0.0061)	0.9590	0.3617	0.0501
4	0.5	−0.022	Ind	−0.0220 (−0.0259, −0.0181)	0.8787	0.9823	
			AR-1	−0.0220 (−0.0259, −0.0181)	0.9443	0.9567	
			CS	−0.0219 (−0.0260, −0.0179)	0.8950	0.9593	
			IND-I	−0.0220 (−0.0290, −0.0147)	0.8630	0.7057	
			Mixed AR-1*	−0.0218 (−0.0257, −0.0181)	0.9584	0.9377	
5	0.99	−0.01	Ind	−0.0102 (−0.0202, −0.0007)	0.4337	0.6403	**0.5730**
			AR-1	−0.0101 (−0.0149, −0.0053)	0.9373	0.3323	**0.0620**
			CS	−0.0101 (−0.0154, −0.0051)	0.4787	0.7840	**0.5120**
			IND-I	−0.0101 (−0.0154, −0.0051)	0.4810	0.7790	**0.5153**
			Mixed AR-1*	−0.0096 (−0.0141, −0.0049)	0.9306	0.3024	**0.0642**
6^**§**^	**0.99**	**−0.022**	**Ind**	**−0.0217 (−0.0317, −0.0117)**	**0.4247**	**0.8300**	
			**AR-1**	**−0.0221 (−0.0266, −0.0174)**	**0.9420**	**0.9017**	
			**CS**	**−0.0221 (−0.0273, −0.0169)**	**0.4817**	**0.9900**	
			**IND-I**	**−0.0221 (−0.0274, −0.0168)**	**0.4787**	**0.9890**	
			Mixed AR-1*	**−0.0215 (−0.0264, −0.0171)**	**0.9254**	**0.8657**	

Covariance matrix structures include independence (Ind), autoregressive (AR-1) & compound symmetry (CS). IND-I corresponds to the model with independence covariance structure and a mouse indicator (fixed-effects model). Mixed AR-1 corresponds to the mixed-effects model with random intercept.

*The percentage of datasets for which the model did not converge was 1.3, 1.8, 5, 8.5, 25, 34.2 for Scenario 1, 2, 3, 4, 5, 6, respectively. For the scenarios for type I error evaluation, the associated percentages were 0.7, 5 and 22 for ρ of 0, 0.5 and 0.99, respectively.

**Type I error is derived from corresponding scenarios with ${\beta }_{2}$ = 0.

^§^Scenarios in bold face reflect parameter values actually observed in the experiment.

**Table 4:** Results of simulation study for the SHP2 inhibition experiment with 10 mice per group and 7 measurements per mouse^30^.

Scenario	ρ	True ${\beta }_{2}$	Model	Mean estimated ${\beta }_{2}$ (IQR)	Coverage	Power	Type I error**
1	0	−0.008	Ind	−0.0080 (−0.0118, −0.0041)	0.9477	0.3020	0.0520
			AR-1	−0.0080 (−0.0117, −0.0041)	0.9503	0.2907	0.0483
			CS	−0.0080 (−0.0117, −0.0041)	0.9320	0.3180	0.0590
			IND-I	−0.0082 (−0.0145, −0.0020)	0.9540	0.1347	0.0543
			Mixed AR-1*	−0.0079 (−0.0115, −0.0042)	0.9659	0.2638	0.0385
2	0	−0.015	Ind	−0.0150 (−0.0187, −0.0114)	0.9510	0.7690	
			AR-1	−0.0150 (−0.0187, −0.0114)	0.9550	0.7593	
			CS	−0.0150 (−0.0187, −0.0113)	0.9410	0.7783	
			IND-I	−0.0151 (−0.0213, −0.0090)	0.9570	0.3620	
			Mixed AR-1*	−0.0148 (−0.0186, −0.0110)	0.9605	0.7125	
3	0.5	−0.008	Ind	−0.0079 (−0.0123, −0.0034)	0.8967	0.3323	0.1067
			AR-1	−0.0079 (−0.0123, −0.0035)	0.9490	0.2187	0.0537
			CS	−0.0078 (−0.0125, −0.0033)	0.9090	0.2780	0.0907
			IND-I	−0.0077 (−0.0151, −0.0000)	0.8717	0.2063	0.1150
			Mixed AR-1*	−0.0076 (−0.0118, −0.0033)	0.9564	0.1891	0.0609
4	0.5	−0.015	Ind	−0.0149 (−0.0195, −0.0104)	0.8947	0.7220	
			AR-1	−0.0150 (−0.0196, −0.0103)	0.9500	0.6040	
			CS	−0.0150 (−0.0198, −0.0101)	0.9143	0.6510	
			IND-I	−0.0150 (−0.0225, −0.0075)	0.8853	0.4163	
			Mixed AR-1*	−0.0147 (−0.0194, −0.0101)	0.9511	0.5772	
5^**§**^	**0.96**	**−0.008**	**Ind**	**−0.0083 (−0.0157, −0.0010)**	**0.6522**	**0.4582**	**0.3427**
			**AR-1**	**−0.0082 (−0.0128, −0.0037)**	**0.9507**	**0.2534**	**0.0523**
			**CS**	**−0.0082 (−0.0134, −0.0032)**	**0.6776**	**0.5605**	**0.3047**
			**IND-I**	**−0.0082 (−0.0136, −0.0031)**	**0.6742**	**0.5462**	**0.3093**
			Mixed AR-1*	**−0.0076 (−0.0119, −0.0032)**	**0.9280**	**0.2139**	**0.0597**
6	0.96	−0.015	Ind	−0.0148 (−0.0222, −0.0073)	0.6547	0.6523	
			AR-1	−0.0148 (−0.0192, −0.0103)	0.9413	0.6230	
			CS	−0.0148 (−0.0198, −0.0099)	0.6847	0.8423	
			IND-I	−0.0148 (−0.0200, −0.0097)	0.6787	0.8220	
			Mixed AR-1*	−0.0144 (−0.0190, −0.1000)	0.9272	0.5857	

Covariance matrix structures include independence (Ind), autoregressive (AR-1) & compound symmetry (CS). IND-I corresponds to the model with independence covariance structure and a mouse indicator (fixed-effects model). Mixed AR-1 corresponds to the mixed-effects model with random intercept.

*The percentage of datasets for which the model did not converge was 20.7, 24.7, 32.6, 37, 48, 50 for Scenario 1, 2, 3, 4, 5, 6, respectively. For the scenarios for type I error evaluation, the associated percentages were 18.5, 32 and 44 for ρ of 0, 0.5 and 0.96, respectively.

**Type I error is derived from corresponding scenarios with ${\beta }_{2}$ = 0.

^§^Scenarios in bold face reflect parameter values actually observed in the experiment.

### Sensitivity analysis

**Figure 1:**
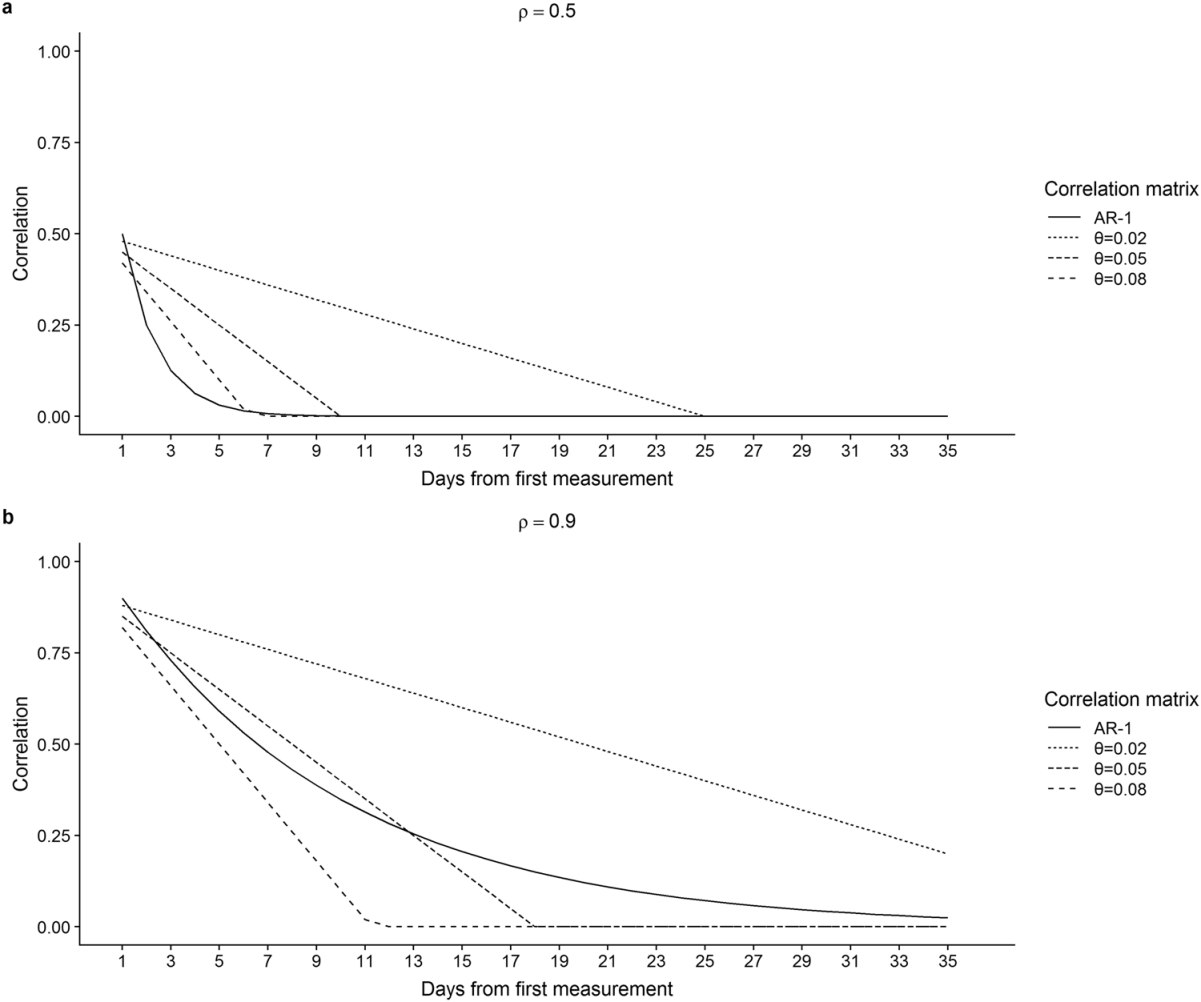
Correlation between measurement at each time point and first measurement, for four correlation matrices. (**a**) ρ = 0.5 (**b**) ρ = 0.9.

Judging from the results above, the AR-1 model was favoured in our main simulation study. This may have been partly due to the fact that in the data generating mechanism we used an AR-1 correlation structure as well. However, when data were generated under autocorrelations not exactly AR-1, unbiased estimates of the interaction effect were obtained under all scenarios but the type I error rates were inflated and the coverage rates were deflated, depending on the magnitude of the misspecification of the variance-covariance matrix (Table 5, Fig. 1). If the alternative correlation pattern used to generate data was not very different from AR-1, which was true for specific numbers of measurements per mouse and parameter values θ and ρ that defined the association between two measurements on consecutive days, then its performance was acceptable under all examined scenarios. Nonetheless, we observed larger type I error and smaller coverage with larger misspecification of the association between repeated measurements using the AR-1 model. When data generated under correlation decreasing linearly with time were analysed with compound symmetry or independent correlation structures, coverage and type I error were severely non-nominal (data not shown).

**Table 5:** Results from simulation study with data generated under a linearly decreasing correlation structure*.

Experiment	ρ	True ${\beta }_{2}$	Coverage			Type I error**		
			θ=0.02	θ=0.05	θ=0.08	θ=0.02	θ=0.05	θ=0.08
Buoninfante *et al*.^28^^§^	0.5	−0.002	0.8370	0.9147	0.9417	0.1627	0.0847	0.0597
		−0.0096	0.8387	0.9167	0.9460			
	**0.85**	−0.002	0.9110	0.9250	0.9457	**0.0873**	**0.0613**	**0.0447**
		**−0.0096**	**0.9107**	**0.9300**	**0.9547**			
Boshuizen *et al*.^§,^^29^	0.5	−0.01	0.8243	0.9093	0.9373	0.1710	0.0923	0.0683
		−0.022	0.8353	0.9137	0.9407			
	**0.99**	−0.01	0.9307	0.9257	0.9563	**0.0757**	**0.0743**	**0.0503**
		**−0.022**	**0.9377**	**0.9197**	**0.9530**			
Mainardi *et al*.^30^^§^	0.5	−0.008	0.9100	0.9250	0.9330	0.0890	0.085	0.070
		−0.015	0.9120	0.9237	0.9353			
	**0.96**	**−0.008**	**0.9727**	**0.9367**	**0.9160**	**0.0263**	**0.053**	**0.079**
		−0.015	0.9660	0.9410	0.9190			

^*^Variance-covariance matrix with non-diagonal elements ρ-θ*Δ(t) where Δ(t) is the time difference between measurements (see paragraph on sensitivity analysis in methods section).

**Type I error is derived from corresponding scenarios with ${\beta }_{2}$ = 0.

^§^Scenarios in bold face reflect parameter values actually observed in the experiment.

## Discussion

We demonstrate that in tumour growth experiments unbiased estimates of the difference in tumour growth rates by treatment group, i.e., the interaction term, and confidence intervals with nominal coverage can be obtained using a linear regression model with an autoregressive (AR-1) variance-covariance structure. These conclusions hold for a wide range of realistic scenarios based on three previous experiments with small numbers of mice and highly correlated longitudinal measurements. Although we recommend this method, which is relatively simple and implemented in all major statistical software packages, results need to be interpreted with care because type I errors could be somewhat inflated due to misspecification of the covariance structure.

Longitudinal data is usually analysed using mixed-effects models, where repeated measurements are nested within subjects. Many researchers apply random intercept only models, where the intercept is the only parameter that varies between subjects while all other parameters, e.g., the time slope, are fixed. However, in our simulation study we experienced that such models do not always converge. In some of the evaluated scenarios, we detected non-convergence problems in up to 50% of the simulated datasets. Since experimental mice are genetically identical and share the same environment, there is a small variability of the log-volume at the time of randomization between mice suggesting very similar estimations of individual mouse intercepts.

Guerin and Stroup^22^ performed a simulation study on repeated measures data and analysed them with random intercept only models with several variance-covariance matrix structures. Their study is very similar to ours in terms of the research goal, and it was the only study that evaluated properties of the interaction term. Exploring type I error rates, convergence and several model selection criteria, they concluded that the Kenward-Roger correction should be used with small sample sizes. However, they also experienced non-convergence problems with the random intercept model. The authors proposed dropping the between subject random intercept if its variance is approximately zero, i.e., using a model with only fixed parameters.

Wang and Goonewardene^23^ explored the use of random intercept only models for repeated measures data in animal experiments and recommended a model with the first order ante dependence (ANTE(1)) covariance structure, which allowed for unequal variances over time and unequal correlations and covariance among different pairs of measurements. This recommendation was based on small sample behaviour of typical animal experiments conducted in animal health and agricultural settings however where animals are not identical, e.g. steers or cows.

Using example data on BT-20 human breast tumour in nude mice, Heitjan *et al*.^15^ compared the most commonly used statistical methods to analyse tumour growth experiments *in vivo*, including ANOVA, t-test, and Mann-Whitney methods. They concluded that these approaches could be misleading due to severely inflated type I errors. Instead, multivariate models, like MANOVA or a random effects model with AR-1 covariance structure should be used, because they retained the nominal type I error rates in various sample sizes, achieving also reasonable levels of statistical power.

Interesting approaches were developed by Zhao *et al*.^39^ to model tumour profiles of mice that had an almost total tumour regression due to initial efficiency of treatment followed by a re-growth phase, and by Laajala *et al*.^25^ to distinguish between growing and poorly growing tumours in mice experiments, thus to model the tumour heterogeneity. There are also other studies that have evaluated small sample properties of methods for the analysis of correlated data, but these were focused on hierarchical data instead of longitudinal data^40,41^. McNeish and Stapleton^18^ compared twelve methods, including Bayesian alternatives, for analysing hierarchical data with small to moderate sample sizes. Using the results from a real life study from educational psychology, they conducted a broad and comprehensive simulation study to assess the statistical properties of the regression coefficient estimates as well as those of the variance component estimates. Even with less than ten clusters and less than 14 observations per cluster, some methods resulted in efficient parameter estimates. Simulations showed that mixed-effects models estimated with Markov chain Monte Carlo algorithm and an inverse gamma prior performed well with such small samples. With a half-Cauchy prior for a slightly larger number of observations per cluster, up to 34, a somewhat better performance could be achieved. The study also showed that fixed-effects models performed well and should be considered as an alternative approach in similar studies. However, the investigation was not focused on longitudinal data but clustered data where each individual had only one measurement and individuals whose outcome could be correlated were clustered together.

Pekar and Brabac^42^ compared generalized least squares regression with mixed-effects models using five data examples from behavioural research, including longitudinal data, and suggested that the former was an effective alternative method for analysing correlated data in that field and when the random effects were not of the researcher’s particular interest.

Our study has a number of strengths. First of all, we use real data from previous experiments in order to understand the characteristics of the methods in relevant circumstances. Our simulations are also based on these data and therefore reflect realistic scenarios, tailored to mice experiments. Moreover, the methods we investigate are very simple and therefore accessible to non-statisticians. Finally, the methods are implemented in most statistical software.

Our study has also several limitations. (1) We assume that log-transformed tumour volume is linearly associated with time. This assumption seems adequate, since tumours commonly grow slowly during the first days of treatment, before they become resistant, and then grow much faster. However, tumour volume may sometimes initially decrease due to treatment efficacy and eventually increase when the tumour becomes resistant to the treatment. Even in this case, a linear approximation of the growth patterns should allow detection of substantial group differences. The alternative, namely using complex flexible relationships has the drawback that it involves many parameters resulting in tests with low power. (2) We generate equal numbers of tumour volume measurements for all mice in a study. This means that we do not allow for the fact that some mice are sacrificed before the end of the study, i.e. when they suffer too much or their tumour exceeds a threshold size. We assume that using these additional data, which are not available in real experiments, does not add any new information about the tumour growth over time. Therefore, it does not influence the point estimate of tumour growth, although power might be slightly overestimated. The comparison of different methods based on the same generated data is unlikely to be affected. (3) We generate data using the AR-1 variance-covariance matrix although in reality other correlation patterns for longitudinal data are possible. We perform sensitivity analyses generating data under a covariance matrix where the correlation between measurements within mice decays linearly with time, using three different scenarios. The results show that a misspecification of the covariance matrix might have an effect on the inference but not on the estimate of the interaction effect. Although the AR-1 model performs best in our simulation, its performance depends on the magnitude of the misspecification, as well as on the true value of correlation between measurements. High type I errors lead to more false positives results and, therefore, results should be interpreted with caution, particularly if p-values are borderline significant. (4) Finally, our models, as those of others^43–45^, do not include a main effect of treatment which results in a slightly higher power level for the interaction term between time and treatment in comparison to a model which includes this effect (data not shown). The omission of the main effect is reasonable in mice experiments since any difference between treatment groups at the time of randomization is due to chance. Mice are genetically identical, they share the same environment and are randomized to treatment groups when they reach similar tumour volume. I.e., there are no specific reasons why the average tumour volume between the treatment groups at the beginning of the study could differ.

Our results demonstrate that the generalized least squares regression (GLS) with an autoregressive (AR-1) variance-covariance matrix provides efficient and unbiased results as well as nominal coverage and type I error for a broad range of realistic scenarios and for sample sizes as low as 6 mice per group and a moderate number of measurements. The method is, however, somewhat sensitive to misspecification of the correlation structure, with moderately sub-nominal coverage and type I error if the true underlying correlation structure is not too different from AR-1. The use of correlation structures such as compound symmetry or independence when the true underlying correlation structure is similar or close to AR-1 results in severely inflated type I error. The AR-1 model with random intercept can lead to convergence problems. These methods should therefore not be used in mice experiments on tumour growth.

Although we focused on one particular outcome, the recommended model can be implemented to evaluate other outcomes studied in preclinical animal experiments. For example, as recently reported by Zhao *et al*.^14^, a repeated measurements design is common in studies on body weight change over time collected in mice experiments. Authors reviewed 58 manuscripts assessing this outcome and found that less than half of the studies were analysed with a method that fully utilized all collected data. In addition, the authors stressed the importance to incorporate statistical methods for repeated measurements when multiple measurements per mouse are available. Therefore, we hope our recommended model will be considered to study various outcomes collected in preclinical animal experiments.
